# Assessment of Clinical Palliative Care Trigger Status vs Actual Needs Among Critically Ill Patients and Their Family Members

**DOI:** 10.1001/jamanetworkopen.2021.44093

**Published:** 2022-01-20

**Authors:** Christopher E. Cox, Deepshikha Charan Ashana, Krista L. Haines, David Casarett, Maren K. Olsen, Alice Parish, Yasmin Ali O’Keefe, Mashael Al-Hegelan, Robert W. Harrison, Colleen Naglee, Jason N. Katz, Allie Frear, Elias H. Pratt, Jessie Gu, Isaretta L. Riley, Shirley Otis-Green, Kimberly S. Johnson, Sharron L. Docherty

**Affiliations:** 1 Division of Pulmonary and Critical Care Medicine, Duke University School of Medicine, Durham, North Carolina; 2 Program to Support People and Enhance Recovery (ProSPER), Duke University, Durham, North Carolina; 3 Division of Trauma and Critical Care and Acute Care Surgery, Department of Surgery, Duke University, Durham, North Carolina; 4 Section of Palliative Care and Hospice Medicine, Duke University, Durham, North Carolina; 5 Department of Biostatistics and Bioinformatics, Duke University, Durham, North Carolina; 6 Durham Center of Innovation to Accelerate Discovery and Practice Transformation, Durham Veterans Affairs Health Care System, Durham, North Carolina; 7 Department of Anesthesiology, Duke University, Durham, North Carolina; 8 Division of Cardiology, Duke University School of Medicine, Durham, North Carolina; 9 Collaborative Caring, Toluca Lake, California; 10 Division of General Internal Medicine, Duke University School of Medicine, Durham, North Carolina; 11 Duke University School of Nursing, Durham, North Carolina

## Abstract

**Importance:**

Palliative care consultations in intensive care units (ICUs) are increasingly prompted by clinical characteristics associated with mortality or resource utilization. However, it is not known whether these triggers reflect actual palliative care needs.

**Objective:**

To compare unmet needs by clinical palliative care trigger status (present vs absent).

**Design, Setting, and Participants:**

This prospective cohort study was conducted in 6 adult medical and surgical ICUs in academic and community hospitals in North Carolina between January 2019 and September 2020. Participants were consecutive patients receiving mechanical ventilation and their family members.

**Exposure:**

Presence of any of 9 common clinical palliative care triggers.

**Main Outcomes and Measures:**

The primary outcome was the Needs at the End-of-Life Screening Tool (NEST) score (range, 0-130, with higher scores reflecting greater need), which was completed after 3 days of ICU care. Trigger status performance in identifying serious need (NEST score ≥30) was assessed using sensitivity, specificity, positive and negative likelihood ratios, and C statistics.

**Results:**

Surveys were completed by 257 of 360 family members of patients (71.4% of the potentially eligible patient–family member dyads approached) with a median age of 54.0 years (IQR, 44-62 years); 197 family members (76.7%) were female, and 83 (32.3%) were Black. The median age of patients was 58.0 years (IQR, 46-68 years); 126 patients (49.0%) were female, and 88 (33.5%) were Black. There was no difference in median NEST score between participants with a trigger present (45%) and those with a trigger absent (55%) (21.0; IQR, 12.0-37.0 vs 22.5; IQR, 12.0-39.0; *P* = .52). Trigger presence was associated with poor sensitivity (45%; 95% CI, 34%-55%), specificity (55%; 95% CI, 48%-63%), positive likelihood ratio (1.0; 95% CI, 0.7-1.3), negative likelihood ratio (1.0; 95% CI, 0.8-1.2), and C statistic (0.50; 95% CI, 0.44-0.57).

**Conclusions and Relevance:**

In this cohort study, clinical palliative care trigger status was not associated with palliative care needs and no better than chance at identifying the most serious needs, which raises questions about an increasingly common clinical practice. Focusing care delivery on directly measured needs may represent a more person-centered alternative.

## Key Points

**Question:**

Does clinical palliative care trigger status accurately identify individuals with the most serious unmet palliative care needs?

**Findings:**

In this cohort study including 257 dyads (1 patient in an intensive care unit [ICU] and 1 family member of each patient), there was no significant difference in self-reported palliative care needs between those with and without a clinical trigger (median Needs at the End-of-Life Screening Tool scores, 21.0 vs 22.5). Clinical triggers’ 45% sensitivity and 55% specificity suggested that they were no better than chance at identifying serious needs.

**Meaning:**

The findings suggest that using clinical trigger status to prompt palliative care consultation in ICU settings may be an inefficient use of this limited resource; incorporating direct measures of unmet need in care may be a promising alternative strategy.

## Teaser

This cohort study assesses whether higher levels of family member–reported palliative care needs are observed among those whose critically ill loved ones meet a clinical palliative care trigger compared with those who do not meet such a trigger.

## Introduction

Critical illness is deeply challenging for the millions of persons who experience it in intensive care units (ICUs) each year.^1^ Patients often experience serious symptoms as well as depersonalized death in this technology-focused setting.^2,3^ Their family members commonly report severe stress, decisional conflict related to goals of care for their loved ones, and feeling unsupported in a complex environment staffed by multiple clinicians.^4,5,6,7,8^

Palliative care is a core attribute of high-quality ICU care because it aims to improve or maintain quality of life and alleviate symptoms by addressing unmet needs of those with serious illness.^9,10^ However, ICU-based palliative care is highly variable because there is no consensus on how to identify those most likely to benefit, deliver the appropriate level of primary or specialist palliative care, or measure its association with person-centered outcomes.^11,12,13,14^ Although the consultative model of specialist palliative care has dominated practice for decades,^15^ intensivists order these consultations infrequently and often late in the course of a patient’s stay in an ICU.^16^ To increase the presence of palliative care specialists in ICUs, professional societies and experts have recommended protocols that trigger specialist consultation based on clinical characteristics associated with death or resource utilization, such as advanced cancer or cardiac arrest.^15,17,18^

Although trigger status–based protocols have been increasingly implemented in a variety of clinical settings by health care systems worldwide,^19^ it is not known whether such clinical and resource utilization triggers are acceptable proxy measures of palliative care needs such as symptoms, decisional conflict, spiritual concerns, or misalignment of values and treatments. An additional concern about these protocols is the dramatic imbalance between the enormous number of persons with a trigger criterion present—more than 2 million patients treated in ICUs each year—and the fewer than 7600 palliative care specialists in the US.^20,21,22^ Because this resource of palliative care is operationally scarce and geographically inconsistent, it is critical that palliative care specialists be appropriately matched with those patients and family members most likely to benefit and optimally integrated with the primary care teams caring for them in both outpatient and inpatient settings.^23^

The primary aim of this prospective cohort study was to test the hypothesis that higher levels of family member–reported palliative care needs would be observed among those whose critically ill loved ones met a clinical palliative care trigger compared with those who did not meet such a trigger. We also assessed the performance characteristics of the presence of a clinical palliative care trigger for identifying the most serious needs.

## Methods

### Study Design, Setting, and Participants

We conducted an observational cohort study of 257 dyads (1 patient in an ICU and 1 family member of each patient) in 6 adult medical and surgical ICUs in 1 academic hospital and 1 large community hospital in the Duke University Health System between January 2019 and September 2020. The study was approved by Duke University Institutional Review Board. All participants or their legal representatives provided written informed consent. We followed the Strengthening the Reporting of Observational Studies in Epidemiology (STROBE) reporting guideline.

To construct a cohort of seriously ill persons, we included consecutive patients 18 years or older who received mechanical ventilation for 48 hours or longer. Exclusion criteria were the presence of decisional capacity, given our focus on surrogate decision makers; clinician expectation of death within 24 hours; imminent plan for comfort care; imprisonment; and lack of an available family member. Previous palliative care consultation during the admission was not a criterion for exclusion. We enrolled 1 adult family member per patient who self-identified as the individual most involved in the patient’s care. Family members were excluded if they lacked sufficient English fluency to complete study surveys. We aimed to obtain informed consent after 2 to 3 full days of ICU care to allow time for the completion of an ICU team–led family meeting.^24^

### Data Collection and Outcomes

Family members completed electronic surveys through secure weblinks automatically emailed or sent by SMS text from the study web-based survey data system (REDCap; Vanderbilt University) at the time of consent.^25^ Clinical data were abstracted manually by study staff from the electronic medical records.

#### Family-Reported Measures

The primary outcome was the Needs at the End-of-Life Screening Tool (NEST).^26,27^ The NEST had been previously adapted to the ICU setting in a series of validation studies that included item derivation, formal cognitive testing with ICU family members, comparison with standard symptom and communication instruments including those used in this study, and evaluation of responsiveness.^25^ The 13-item NEST (score range, 0 [no need] to 130 [highest need]) assesses needs in all 8 domains of palliative care quality, including structure and processes, physical symptoms, psychological symptoms, social support, spiritual and cultural aspects of care, end-of-life care, and ethical aspects of care.^28^

Secondary outcomes included family report of goal-concordant care, a dichotomous outcome that compares actual with preferred treatment (yes indicates that actual treatment provided is identical to preferred treatment; no indicates that actual treatment differs from preferred treatment),^29^ and the Quality of Communication scale summary item (score range, 0 [worst] to 10 [best]).^30^ We quantified depression symptoms using the 9-item Patient Health Questionnaire (PHQ-9; score range, 0 [lowest] to 27 [highest]),^31^ anxiety symptoms with the Generalized Anxiety Disorder 7-item scale (GAD-7; score range, 0 [lowest] to 21 [highest]),^32^ and posttraumatic stress disorder symptoms with the Posttraumatic Stress Syndrome inventory (PTSS; score range, 10 [lowest] to 70 [highest]).^33^ We administered the Interpersonal Processes of Care instrument’s patient-centered decision-making, eliciting concerns, and racial and economic discrimination domains (domain score range, 0 [worst] to 5 [best]).^34^ We administered Likert-scaled items to measure participants’ relationship with the ICU attending physician, expectation for patient survival, and financial distress.^35^

#### Clinical Variables

The primary exposure was any of 9 clinical triggers for palliative care consultation present within the first 48 hours of ICU admission. These clinical triggers, addressing both acute and chronic processes and described in a body of research spanning decades,^11,15,19,20,36^ included cardiac arrest; advanced cancer; dementia; critical acute neurologic condition; residence in a long-term acute care facility, skilled nursing facility, or inpatient rehabilitation facility; 3 or more limitations in baseline activities of daily living; 2 or more hospital admissions or 1 or more ICU admissions within 3 months; and worsening organ dysfunction (ie, increase between 24 and 48 hours after ICU admission in Sepsis-related Organ Failure Assessment score)^37^; details can be found in eTable 1 in the Supplement. We also recorded insurance status, comorbidities, diagnoses, Acute Physiology and Chronic Health Evaluation II illness severity scores,^38^ code status, receipt of palliative care consultation, length of stay, and discharge disposition. Race, ethnicity, and gender were self-reported by participants.

### Statistical Analysis

Power calculations to test the primary hypothesis were based on a 1-sided test with a type I error of .025.^39^ Using an SD of 15 for the NEST,^25^ a comparison of 100 dyads with triggers present with 100 dyads with triggers absent was estimated to provide 80% power to identify a difference in NEST score less than 6 points (half the range of a single item).

We compared characteristics and outcomes by trigger status using Wilcoxon rank sum, χ^2^, Kruskal-Wallis, or Fisher exact tests as appropriate. The performance of trigger status for identifying serious need was calculated from a 2 × 2 table using sensitivity, specificity, positive and negative predictive values, accuracy, positive and negative likelihood ratios, and C statistics (details in eTable 2 in the Supplement). Previous use of the NEST defined threshold scores for each item (range, ≥3 to ≥6; mean, ≥5).^27^ Therefore, serious need was defined as a total NEST score of 30 or higher, reflecting 3 possible scenarios of clinical significance that could prompt clinician concern: an average item score above the threshold of 5 or greater in a majority of domains, extremely high needs (NEST score, 8-10) in a few of the 8 palliative care quality domains sampled, or moderate needs (NEST score, 3-5) for nearly all domains. We also examined NEST score ranges from 10 to 50 in sensitivity analyses.

## Results

### Patient and Family Member Characteristics

Among 1179 consecutive patients screened, 360 potentially eligible patient–family member dyads were approached for enrollment; 262 (72.7%) provided informed consent at a mean (SD) of 4.8 (4.3) days after ICU admission, and 257 dyads (71.4%; 514 participants) had complete data for inclusion in analyses. Patients were generally middle-aged; the median age of participants was 58 years (IQR, 46-68 years). Of the 257 patients in the cohort, 131 (51.0%) were men; 86 (33.5%) self-identified as Black or African American, and 147 (57.2%) self-identified as White. Care was provided for 148 patients (57.6%) in medical ICUs and for 109 patients (42.4%) in surgical ICUs (Table 1). Of the 257 family members in the dyads, 197 (76.7%) were female; 114 (44.4%) were the spouse or partner of the patient; and 124 (48.6%) had received at least some college education. Although it was not a criterion for study exclusion, no dyad had received a palliative care consultation at the time of survey completion.

**Table 1.** Patient and Family Member Characteristics and Outcomes by Palliative Care Trigger Status

	Patients				Family members			
	No. (%)			*P* value	No. (%)			*P* value
	Trigger absent (n = 142)	Trigger present (n = 115)	Total (N = 257)		Trigger absent (n = 142)	Trigger present (n = 115)	Total (N = 257)	
**Characteristic**								
Age, median (IQR), y	54.5 (42.9-67.0)	62.0 (52.0-69.0)	58.0 (46.0-68.0)	.007^a^	54.0 (44.0-62.0)	53.0 (43.0-62.0)	54.0 (44.0-62.0)	.72^a^
Gender								
Male	74 (52.1)	57 (49.6)	131 (51.0)	.69^b^	35 (24.6)	24 (20.9)	59 (23.0)	.45^c^
Female	68 (47.9)	58 (50.4)	126 (49.0)		107 (75.4)	90 (78.3)	197 (76.7)	
Transgender	0	0	0		0	1 (0.9)	1 (0.4)	
Race								
American Indian or Alaska Native	3 (2.1)	3 (2.6)	6 (2.3)	.58^c^	2 (1.4)	3 (2.6)	5 (1.9)	.96^c^
Asian	3 (2.1)	2 (1.7)	5 (1.9)		3 (2.1)	3 (2.6)	6 (2.3)	
Black or African American	48 (33.8)	38 (33.0)	86 (33.5)		44 (31.0)	39 (33.9)	83 (32.3)	
Native Hawaiian or Pacific Islander	0	0	0		1 (0.7)	0	1 (0.4)	
White	78 (54.9)	69 (60.0)	147 (57.2)		87 (61.3)	67 (58.3)	154 (59.9)	
Other^d^	10 (7.0)	3 (2.6)	13 (5.1)		4 (2.8)	2 (1.7)	6 (2.3)	
>1 Race	0	0	0		1 (0.7)	1 (0.9)	2 (0.8)	
Not reported	0	0	0		5 (3.5)	5 (4.3)	10 (3.9)	
Hispanic or Latinx ethnicity	4 (2.8)	2 (1.7)	6 (2.3)	.69^c^	6 (4.2)	2 (1.7)	8 (3.1)	.30^c^
Insurance status								
Commercial	60 (42.3)	36 (31.3)	96 (37.4)	.05^c^	NA	NA	NA	
Medicare	42 (29.6)	52 (45.2)	94 (36.6)		NA	NA	NA	
Medicaid	23 (16.2)	20 (17.4)	43 (16.7)		NA	NA	NA	
None	13 (9.2)	4 (3.5)	17 (6.6)		NA	NA	NA	
Other	4 (2.8)	3 (2.6)	7 (2.7)		NA	NA	NA	
Patient is spouse or partner	NA	NA	NA		69 (48.6)	45 (39.1)	114 (44.4)	.13^b^
Employed	NA	NA	NA		97 (68.3)	79 (68.7)	176 (68.5)	.95^b^
Some college or less	NA	NA	NA		66 (46.8)	58 (50.9)	124 (48.6)	.52^b^
Financial distress^e^	NA	NA	NA		72 (50.7)	56 (48.7)	128 (49.8)	.75^b^
**Outcome**								
ICU								
Medical	58 (40.8)	37 (32.2)	95 (37.0)	.06^b^	NA	NA	NA	
Neurologic	23 (16.2)	34 (29.6)	57 (22.2)		NA	NA	NA	
Surgical	29 (20.4)	20 (17.4)	49 (19.1)		NA	NA	NA	
Cardiac	16 (11.3)	17 (14.8)	33 (12.8)		NA	NA	NA	
Medical-surgical community	13 (9.2)	7 (6.1)	20 (7.8)		NA	NA	NA	
Cardiothoracic surgery	3 (2.1)	0	3 (1.2)		NA	NA	NA	
ICU admission source								
Transfer from outside hospital	68 (47.9)	50 (43.5)	118 (45.9)	.20^b^	NA	NA	NA	
Emergency department	50 (35.2)	52 (45.2)	102 (39.7)		NA	NA	NA	
Postoperative	16 (11.3)	6 (5.2)	22 (8.6)		NA	NA	NA	
Hospital unit	8 (5.6)	7 (6.1)	15 (5.8)		NA	NA	NA	
ICU admission diagnosis								
Acute respiratory failure	60 (42.3)	31 (27.0)	91 (35.4)	.03^b^	NA	NA	NA	
Acute neurologic event or altered mental status	28 (19.7)	34 (29.6)	62 (24.1)		NA	NA	NA	
Shock	23 (16.2)	31 (27.0)	54 (21.0)		NA	NA	NA	
Trauma or postoperative care	28 (19.7)	15 (3.0)	43 (16.7)		NA	NA	NA	
Renal failure	2 (1.4)	2 (1.7)	4 (1.6)		NA	NA	NA	
Liver failure	1 (0.7)	2 (1.7)	3 (1.2)		NA	NA	NA	
APACHE II score, median (IQR), U	22.0 (17.0-27.0)	23.0 (20.0-29.0)	22.0 (18.0-28.0)	.03^a^	NA	NA	NA	
Chronic medical comorbidities, median (IQR), No.	1.0 (0.0-2.0)	2.0 (1.0-3.0)	1.0 (1.0-3.0)	.01^a^	NA	NA	NA	
Trigger present								
Multisystem organ failure that worsened >48 h from ICU admission	NA	55 (47.8)	55 (21.4)	NA	NA	NA	NA	
Severe acute neurologic condition	NA	26 (22.6)	26 (10.1)		NA	NA	NA	
≥3 Limitations in activities of daily living	NA	24 (20.9)	24 (9.3)		NA	NA	NA	
Cardiac arrest	NA	24 (20.9)	24 (9.3)		NA	NA	NA	
Advanced cancer	NA	22 (19.1)	22 (8.6)		NA	NA	NA	
≥1 ICU admission within 3 mo	NA	22 (19.1)	22 (8.6)		NA	NA	NA	
≥2 Hospital admissions within 3 mo	NA	20 (17.4)	20 (7.8)		NA	NA	NA	
Admitted from post–acute care facility^f^	NA	18 (15.7)	18 (7.0)		NA	NA	NA	
Dementia	NA	3 (2.6)	3 (1.2)		NA	NA	NA	
Total triggers present, median (IQR), No.	NA	2.0 (1.0-2.0)	0.0 (0.0-1.0)		NA	NA	NA	
Mechanical ventilation, median (IQR), d	9.6 (5.2-17.8)	10.1 (6.0-14.4)	9.9 (5.6-16.5)	.99^a^	NA	NA	NA	
ICU stay duration, median (IQR), d	11.5 (6.0-21.0)	12.0 (5.0-20.0)	12.0 (6.0-20.0)	.60^a^	NA	NA	NA	
Hospital stay duration, median (IQR), d	26.0 (16.0-43.0)	25.0 (12.0-38.0)	26.0 (14.0-41.0)	.28^a^	NA	NA	NA	
Palliative care consultation^g^	12 (8.5)	26 (22.6)	38 (14.8)	.002^b^	NA	NA	NA	
Code status during hospitalization								
Full code throughout	102 (71.8)	61 (53.0)	163 (63.4)	.002^b^	NA	NA	NA	
Full code to DNAR	37 (26.1)	48 (41.7)	85 (33.1)		NA	NA	NA	
DNAR throughout	1 (0.7)	6 (5.2)	7 (2.7)		NA	NA	NA	
DNAR to full code	2 (1.4)	0	2 (0.8)		NA	NA	NA	
Discharge disposition								
Home, independent	37 (26.1)	10 (8.7)	47 (18.3)	<.001^b^	NA	NA	NA	
Home with paid health care services	26 (18.3)	5 (4.3)	31 (12.1)		NA	NA	NA	
Post–acute care facility^f^	44 (30.9)	49 (42.6)	93 (36.2)		NA	NA	NA	
Transfer to other hospital	1 (0.7)	2 (1.7)	3 (1.2)		NA	NA	NA	
Hospice^h^	6 (4.2)	8 (7.0)	14 (5.5)		NA	NA	NA	
Died	28 (19.7)	41 (35.7)	69 (26.8)		NA	NA	NA	

Abbreviations: APACHE II, Acute Physiology and Chronic Health Evaluation Illness Severity Score; DNAR, do not attempt resuscitation; ICU, intensive care unit; NA, not applicable.

^a^ Wilcoxon rank sum test.

^b^ χ^2^ Test.

^c^ Fisher exact test.

^d^ Other was provided as an alternative if the rest of the choices were deemed not appropriate by the participant.

^e^ Having little money left after paying bills, having to cut back to pay bills, or having difficulty paying bills no matter what.

^f^ Inpatient rehabilitation facility, skilled nursing facility, or long-term acute care facility.

^g^ No patient-family dyad had an active palliative care consult at the time of study enrollment; all consultations occurred after enrollment.

^h^ Home or inpatient.

### Palliative Care Trigger Status Results

#### Clinical Characteristics

A clinical trigger was present for 115 patients (44.7%); triggers included worsening organ dysfunction in 55 patients (47.8%), severe acute neurologic condition in 26 (22.6%), limitations in 3 or more activities of daily living in 24 (20.9%), cardiac arrest in 24 (20.9%), advanced cancer in 22 (19.1%), 1 or more recent ICU admissions in 22 (19.1%), 2 or more hospital admissions in 20 (17.4%), admission from a facility in 18 (15.7%), and dementia in 3 (2.6%). Compared with patients for whom triggers were absent, those with triggers present were older (median age, 62 years; IQR, 52.0-69.0 years vs 54.5 years; IQR, 42.9-67.0 years), although they were otherwise similar in sociodemographic and clinical characteristics.

#### Trigger Status and Outcomes

There was no difference in either the median total NEST score (21.0; IQR, 12.0-37.0 vs 22.5; IQR, 12.0-39.0; *P* = .52) or the prevalence of serious overall needs (ie, total NEST score ≥30; 38 individuals [33.0%] vs 49 [34.5%]; *P* = .81; Table 2; eFigure in the Supplement) between family members of patients with triggers present and absent, respectively. The groups did not differ in the frequency of family-reported goal-concordant care (90 individuals [78.3%] vs 109 [76.8%]; *P* = .78), communication quality (9.0; IQR, 8.0-10.0 vs 9.0; IQR, 8.0-10.0; *P* > .99), or psychological distress symptoms (all *P* > .32 for PHQ-9 [6.0; IQR, 3.0-12.0 vs 5.0; IQR, 3.0-10.0], GAD-7 [6.0; IQR, 3.0-11.0 vs 6.0; IQR, 2.0-10.0], and PTSS [17.0; IQR, 13.0-26.0 vs 18.5; IQR, 13.0-28.0]), although the family members of patients with triggers present more frequently rated the quality of their relationship with the ICU physician favorably (107 individuals [93.0%] vs 121 [84.3%]; *P* = .049) and less frequently believed patient survival was likely (57 individuals [49.6%] vs 91 [64.1%]; *P* = .02). Length of stay was similar by trigger status in the ICU (12.0; IQR, 5.0-20.0 vs 11.5; IQR 6.0-21.0; *P* = .60) and hospital (25.0; IQR, 12.0-38.0 vs 26.0; IQR, 16.0-43.0; *P* = .28).

**Table 2.** Family Member Outcomes by Trigger Status

Outcome	Outcome by trigger status			
	No. (%)			*P* value
	Trigger absent (n = 142)	Trigger present (n = 115)	Total (N = 257)	
NEST total score, median (IQR)	22.5 (12.0-39.0)	21.0 (12.0-37.0)	22.0 (12.0-37.0)	.52^a^
Serious overall needs^b^	49 (34.5)	38 (33.0)	87 (33.9)	.81^c^
≥1 Serious need^d^	107 (75.4)	93 (80.9)	200 (77.8)	.29^c^
≥1 Need of any severity^e^	139 (97.9)	113 (98.3)	252 (98.1)	.83^c^
Goal-concordant care present	109 (76.8)	90 (78.3)	199 (77.4)	.78^c^
Quality of communication, median (IQR), U	9.0 (8.0-10.0)	9.0 (8.0-10.0)	9.0 (8.0-10.0)	.99^a^
Excellent or good relationship with ICU doctor	121 (84.3)	107 (93.0)	228 (88.7)	.049
Expected patient survival rated as almost certain	91 (64.1)	57 (49.6)	148 (57.6)	.02^c^
PHQ-9 score, median (IQR)	5.0 (3.0-10.0)	6.0 (3.0-12.0)	6.0 (3.0-11.0)	.38^a^
GAD-7 score, median (IQR)	6.0 (2.0-11.0)	6.0 (3.0-11.0)	6.0 (3.0-11.0)	.34^a^
PTSS score, median (IQR)	18.5 (13.0-28.0)	17.0 (13.0-26.0)	18.0 (13.0-27.0)	.32^a^
Interpersonal Processes of Care score, median (IQR)				
Concern domain	5.0 (4.7-5.0)	5.0 (4.3-5.0)	5.0 (4.5-5.0)	.32^a^
Decision-making domain	4.5 (3.5-5.0)	4.3 (3.5-5.0)	4.5 (3.5-5.0)	.79^a^
Discrimination domain	1.0 (1.0-1.0)	1.0 (1.0-1.0)	1.0 (1.0-1.0)	.47^a^

Abbreviations: GAD-7, Generalized Anxiety Disorder 7-Item Scale; ICU, intensive care unit; NEST, Needs at the End-of-Life Screening Tool; PHQ-9, 9-item Patient Health Questionnaire; PTSS, Posttraumatic Stress Syndrome inventory.

^a^ Wilcoxon rank sum test.

^b^ NEST total score of 30 or higher.

^c^ χ^2^ Test.

^d^ At least 1 NEST item score of 5 or higher.

^e^ At least 1 NEST item score higher than 0.

#### Performance Characteristics of Trigger Status for Identifying Serious Needs

The presence of a clinical trigger demonstrated low sensitivity (44.7%; 95% CI, 34.1%-55.3%), specificity (55.2%; 95% CI, 47.8%-62.7%), positive predictive value (33.0%; 95% CI, 24.5%-41.6%), negative predictive value (66.9%; 95% CI, 59.2%-74.6%), and accuracy (51.8%; 95% CI, 45.6%-57.9%) (Figure). Positive likelihood ratios (1.0; 95% CI, 0.7-1.3) and negative likelihood ratios (1.0; 95% CI, 0.8-1.2) suggested that the presence of a trigger did not change the likelihood of serious need. The C statistic (0.50; 95% CI, 0.44-0.57) showed that trigger status was no better than chance at distinguishing dyads with and without serious needs (eTable 3 in the Supplement). Sensitivity analyses revealed little difference in a wide range of NEST scores.

**Figure.**
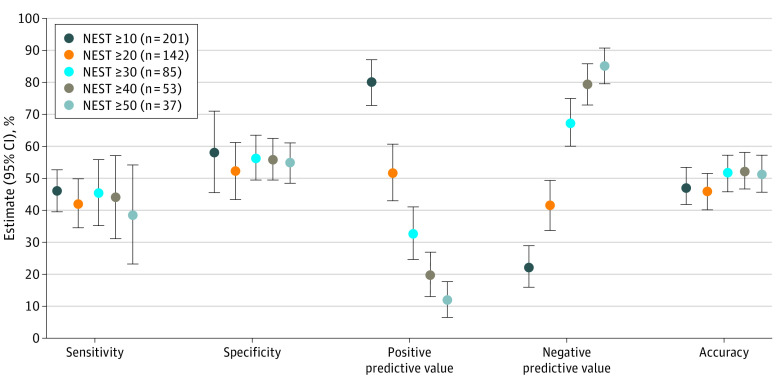
Performance Characteristics of the Presence of Clinical Palliative Care Trigger Characteristics for Identifying Serious Needs Performance characteristics of the presence of a clinical trigger characteristic are shown across a range of definitions for high levels of need based on Needs at the End-of-Life Screening Tool total scores. eTable 2 in the Supplement provides details on the calculation of values, and eTable 3 in the Supplement gives additional results.

#### Association of Needs With Other, Person-Centered Measures

Overall, 87 family members (33.9%) had a serious total burden of needs (ie, total NEST score ≥30), and 200 (77.8%) had at least 1 serious individual need (Table 2). NEST scores were significantly associated with quality of communication (*r* = −0.66; *P* < .001), quality of relationship with the ICU physician (median NEST score, 15.0; IQR, 8.0-24.0 for excellent; 28.0; IQR, 19.0-42.5 for good; 45.0; IQR, 30.0-55.0 for acceptable; and 79.0; IQR, 61.0-95.0 for poor; *P* < .001), psychological distress symptoms (PHQ-9, *r* = 0.19; *P* = .002; GAD-7, *r* = 0.19; *P* = .003; PTSS, *r* = 0.19; *P* = .002), and patient-centeredness scores (eliciting concerns domain *r* = −0.45; *P* < .001; decision-making domain, *r* = −0.59; *P* < .001; racial and economic discrimination domain, *r* = −0.13; *P* = .04) (eTable 4 in the Supplement).

## Discussion

In what we believe is the first comparison of clinical palliative care trigger status with self-reported palliative care needs in an ICU setting, we found that needs did not differ based on the presence or absence of clinical triggers. Furthermore, the presence of a clinical trigger was no better than chance at identifying those with the most serious needs not associated with any patient- and family-centered measure tested and not associated with differences in length of stay. These results may be useful for the development of future models of person-centered ICU-based palliative care delivery.

### Relevance to Past Work

Our findings add to work from Kelley and Bollens-Lunc,^40^ which demonstrated the limitations of administrative database–derived proxy measures of need such as functional dependence or weight loss to screen for palliative care specialist eligibility, and from Wegier et al,^41^ which derived a predictive model for 1-year mortality among inpatients not in the ICU with a threshold value associated with an elevated symptom score. They complement the finding by Creutzfeld et al^42^ that no clinical patient characteristics were associated with neurologic ICU physicians’ perceptions of patient and family member needs. The present study also reinforces past concerns about the disease-centeredness of clinical triggers by demonstrating that neither clinical triggers nor clinical or sociodemographic characteristics were associated with the presence, type, or severity of palliative care needs or with several person-centered outcomes.^15,18,19,43^

### Relevance to Contemporary Care

There is broad agreement that palliative care is most appropriate for those with significant needs, present among 33.9% of participants in this study. However, few methodologies are accepted for either identifying ideal recipients or delivering care within the constraints of palliative care specialists’ small workforce size and geographic inconsistency and intensivists’ limited primary palliative care knowledge.^44^ The patchwork of dissonant approaches that has evolved from this uncertainty likely contributes to the significant practice variability observed within nations, regions, and hospitals.^45,46,47^ This study’s key finding that clinical markers of prognosis and resource utilization may have serious limitations as palliative care screening tools in ICU settings challenges an increasingly popular health care trend that was intended to better standardize care.^19^

### Potential Value of Using Measures of Need in Clinical Care and Research

Our findings also suggest that knowledge of needs may help to improve the identification of those who could benefit most from palliative care, as well as form the foundation of sensible care models in which primary and specialist providers work collaboratively.^48,49^ Although we found that needs were not associated with clinical or sociodemographic characteristics, they can be measured simply and inexpensively using automated email or SMS texting systems as was done in this study. Furthermore, need-based systems can permit visualization by severity, type, or class in a smartphone- or computer-based dashboard and activate specific team providers by text or page based on the need class present.^25^

Our findings suggest that use of a needs-based system could be beneficial in 4 ways. First, knowledge of needs might help ICU teams connect with patients and family members in a more personalized manner.^50^ Incorporating standardized measures of need as actionable data points in care could increase the likelihood that they will be recognized and addressed by clinicians who may be more habitually focused on mechanical ventilator settings or vasopressor doses, given their prominence in electronic health record systems. This recognition could enhance the consistency of person-centered, humane care in the technology-focused ICU setting.^51,52^

Second, the recognition of specific needs could help to more clearly define roles for interprofessional providers and promote the use of adjunctive supportive care interventions, thereby broadening access to palliative care.^49,53^ Chaplains could be called on to assist those with spiritual needs, ICU nurses and respiratory therapists could address patient comfort needs such as pain or breathlessness, and financial counselors and social workers could address financial stress or social support needs.^54,55,56,57,58^ Digital interventions, such as adaptive coping mobile apps or decisional support tools, could also be deployed inexpensively to address stress and decisional conflict.

Third, need-based systems could provide a way to integrate palliative care specialists more sensibly and acceptably within ICU care, thus mitigating many ICU clinicians’ concerns about loss of autonomy.^43,59^ Specialist activation could be focused on the “true positives”—those patients and family members with particularly complex needs at baseline such as needs for decisional support and information, which are most clearly linked to clinician-family interactions in the ICU; specific individual needs of high severity; or demonstration of need that increases over time despite the ICU team’s best efforts. Collaboration could be further enhanced by linking specific needs to team strengths, such as ICU teams’ knowledge of what to expect from critical illness and palliative care specialists’ expertise in symptom management, and by facilitating the prioritization of care to those with the most serious needs.

Fourth, incorporating needs measures in clinical research could help to screen for those patients more likely to demonstrate a response to interventions, highlight novel intervention targets, and add an outcome associated with other, person-centered measures that are directly and temporally relevant to ICU care.^48^

### Limitations

This study has several limitations. Although it was conducted in medical and surgical ICUs among consecutive participants diverse in race and diagnosis, its findings may not be generalizable to other regions and care settings. The time frame of trigger status and need assessment is somewhat arbitrary and involves trade-offs with earlier or later measurement. Although we aimed to assess trigger status and needs relatively early in the ICU course because proactive palliative care is recommended,^60^ study surveys were, on average, completed after nearly 5 days of ICU care—2 days beyond the recommended time by which a formal family meeting should have been conducted.^24^ Furthermore, we designed our measurement approach to reflect what hospitals feasibly implement at a uniform time point, rather than to completely replace the need for clinician-prompted palliative care consultations throughout ICU stays. Although there is no criterion standard need assessment or accepted threshold defining serious need, the adapted NEST addresses all core domains of high-quality palliative care and was associated with multiple person-centered measures. Also, a family member–reported measure may be an imperfect proxy for actual patient needs. Our findings on clinical triggers should not be interpreted as casting doubt on the substantial potential benefits of either palliative care specialist consultation or the concept of triggered consultation in general.^9^

## Conclusions

In this cohort study, we found that the presence of clinical palliative care triggers was not associated with higher levels of unmet palliative care need and was no better than chance in identifying the most serious needs. In contrast, needs were associated with person-centered outcomes and could represent a novel foundation for improving palliative care delivery. These findings raise questions about the increasingly common practice of prompting palliative care specialist consultation in ICU settings based on characteristics associated with death or resource utilization.

## Notes

Supplement. **eTable 1.** Definitions of Clinical Palliative Care Triggers **eTable 2.** Performance Characteristics of the Presence of Clinical Palliative Care Triggers for Identifying Serious Unmet Needs: Additional Information on Analytic Approach **eTable 3.** Performance Characteristics of the Presence of Clinical Palliative Care Triggers for Identifying Unmet Need **eTable 4.** Comparison of Family Member Survey Responses to NEST Scores **eFigure.** Percentage of Participants Reporting Any Level of Individual Need by Trigger Status
