# Supplementary material for: Assessment of Clinical Palliative Care Trigger Status vs Actual Needs Among Critically Ill Patients and Their Family Members
Source: JAMA Netw Open. 2022 Jan 20;5(1):e2144093. doi: 10.1001/jamanetworkopen.2021.44093 (PMC8777568; doi:10.1001/jamanetworkopen.2021.44093)
Supplement: Supplement. — eTable 1. Definitions of Clinical Palliative Care Triggers eTable 2. Performance Characteristics of the Presence of Clinical Palliative Care Triggers for Identifying Serious Unmet Needs: Additional Information on Analytic Approach eTable 3. Performance Characteristics of the Presence of Clinical Palliative Care Triggers for Identifying Unmet Need eTable 4. Comparison of Family Member Survey Responses to NEST Scores eFigure. Percentage of Participants Reporting Any Level of Individual Need by Trigger Status [file jamanetwopen-e2144093-s001.pdf]

Supplementary Online Content

Cox CE, Ashana DC, Haines KL, et al. Assessment of clinical palliative care trigger status vs actual needs among critically ill patients and their family members. *JAMA Netw Open*. 2022;5(1):e2144093. doi:10.1001/jamanetworkopen.2021.44093

eTable 1. Definitions of Clinical Palliative Care Triggers

eTable 2. Performance Characteristics of the Presence of Clinical Palliative Care Triggers for Identifying Serious Unmet Needs: Additional Information on Analytic Approach

eTable 3. Performance Characteristics of the Presence of Clinical Palliative Care Triggers for Identifying Unmet Need

eTable 4. Comparison of Family Member Survey Responses to NEST Scores

eFigure. Percentage of Participants Reporting Any Level of Individual Need by Trigger Status

This supplementary material has been provided by the authors to give readers additional information about their work.

© 2022 Cox CE et al. *JAMA Network Open*.

eTable 1. Definitions of clinical palliative care triggers

Trigger	Definition	Notes
**Organ failure that worsened from the time of ICU admission**	An increase in the Sequential Organ Failure Assessment [SOFA] score between ICU day 1 (0-24 hours) and ICU day 2 (25-48 hours)	Abstracted from medical record. For missing variables 0 was entered (the most optimistic strategy)
**Severe acute neurological injury**	ICU admission prompted by subarachnoid or subdural hemorrhage, ischemic stroke, traumatic brain injury, or status epilepticus (i.e., ICD-10 codes S06.X, I61.4, I61.9, I63.X)	Documented by physician in history and physical as the primary ICU admission diagnosis
**Activities of daily living (ADL) limitations**	≥3 limitations in activities of daily living including bathing, dressing, toileting, transferring, continence, and self-feeding	Abstracted from medical record, including review of admission data, the history and physical note, case management notes, and nursing intake form from time of hospital admission
**Cardiac arrest**	Cardiac arrest immediately preceding and generally prompting ICU admission	Abstracted from medical record, including review of admission data, Code Blue form note, and ICU admission clinician notes
**Advanced cancer**	Currently active and advanced or metastatic solid or liquid cancers (i.e., ICD-10 codes C18.9, C34.9X, C50.91X, C78.X, C79.X). Solid tumors generally included Stage 4 colon, lung, breast cancers or other cancers described as 'metastatic.' For liquid cancers (e.g., leukemia, lymphoma), documentation of 'refractory' or 'advanced' status in treating oncologist's progress note	Abstracted from medical record, including history and physical and progress notes from hematology-oncology service
**Very recent ICU admission**	≥1 ICU admission in the 3 months before the current admission	Abstracted from medical record for admissions within the Duke Health System and history and physical note for admissions from outside the Duke Health System
**Very recent hospital admissions**	≥2 hospital admissions in the 3 months before the current admission	Abstracted from medical record for admissions within the three hospitals within the Duke Health System, as well as the history and physical note for admissions from outside the Duke Health System
**Admission from facility**	Admission to hospital from skilled nursing facility, inpatient rehabilitation facility, or long-term acute care facility	Abstracted from medical record, including review of admission data, the history and physical note, case management notes, and nursing intake form from time of hospital admission
**Dementia**	Alzheimer's, vascular, multi-infarct, or other cause (i.e., ICD-10 codes F01.50, F01.51, F03.90, F03.91, G30.0, G30.1, G30.8, G30.9, G31.83)	Must have been listed as a key issue in the problem list in medical chart or described as 'moderate,' 'severe,' 'serious,' or 'clinically significant' in admission history and physical note

© 2022 Cox CE et al. *JAMA Network Open*.

eTable 2. Performance characteristics of the presence of clinical palliative care triggers for identifying serious unmet needs: additional information on analytic approach

We determined performance characteristics using a 2 x 2 table set up with the NEST score as the gold standard condition of unmet need and the presence of ≥1 trigger as the test condition. We chose to combine all triggers in this way given that this would presumably optimize sensitivity given the application of clinical triggers as a screening, rather than diagnostic test.

	Serious need present (i.e., high NEST score)	Serious need absent (i.e., low NEST score)
≥1 Trigger present	A	B
Trigger absent	C	D

Sensitivity is the true positive rate, or the proportion of true positives (i.e., high NEST score) among all positives (i.e., number who meet a trigger): A / A+C.

Specificity is the true negative rate, or the proportion of true negatives (i.e., low NEST score) among all negatives (i.e., number who do not meet a trigger): D / B+D

Positive predictive value (PPV) is the proportion of those with a high NEST score (i.e., serious needs) who meet a trigger: A / A+B

Negative predictive value (NPV) is the proportion of those with a low NEST score (i.e., less serious needs) who do not meet a trigger: D / C+D

Accuracy is the correct classification rate, or the proportion of those who are either true positives (i.e., high NEST score and therefore had serious needs) and who meet a trigger or true negatives (i.e., low NEST score and therefore did not have serious needs) and who do not meet a trigger: A+B / A+B+C+D

Positive likelihood ratio (PLR) represents the true positive rate (i.e., sensitivity) divided by the false positive rate (1-specificity), or the ratio of the probability of having serious unmet needs when the patient has a trigger divided by the probability of having low needs when the patient does not meet a trigger.

Negative likelihood ratio (NLR) represents the false negative rate (i.e., 1-sensitivity) divided by the true negative rate (specificity), or the ratio of the probability of having serious unmet needs when the patient does not have a trigger divided by the probability of having low needs when a trigger is not present.

C statistic is equal to the area under the curve of a graph of rates of true positives (sensitivity) and false positives (1-specificity).

© 2022 Cox CE et al. *JAMA Network Open*.

eTable 3. Performance characteristics of the presence of clinical palliative care triggers for identifying unmet need [1]

**Characteristic**	**NEST ≥10** n=201 (78.2%)	**NEST ≥20** n=142 (55.3%)	**NEST ≥30** n=85 (33.1%)	**NEST ≥40** n=53 (20.6%)	**NEST ≥50** n=37 (14.4%)
**Trigger status**, *n* (%)					
Absent	110 (54.7)	83 (58.5)	47 (55.3)	30 (56.6)	23 (62.2)
Present	91 (45.3)	59 (41.5)	38 (44.7)	23 (43.4)	14 (37.8)
**Sensitivity**, % (95% CI)	45.3 (38.4, 52.2)	41.6 (33.4, 49.7)	44.7 (34.1, 55.3)	43.4 (30.1, 56.7)	37.8 (22.2, 53.5)
**Specificity**, % (95% CI)	57.1 (44.2, 70.1)	51.3 (42.2, 60.4)	55.2 (47.8, 62.7)	54.9 (48.1, 61.7)	54.1 (47.5, 60.7)
**Positive predictive value (PPV)**, % (95% CI)	79.1 (71.7, 86.6)	51.3 (42.2, 60.4)	33.0 (24.5, 41.6)	20.0 (12.7, 27.3)	12.2 (6.2, 18.2)
**Negative predictive value (NPV)**, % (95% CI)	22.5 (15.7, 29.4)	41.6 (33.4, 49.7)	66.9 (59.2, 74.6)	78.9 (72.2, 85.6)	83.8 (77.7, 89.9)
**Accuracy**, % (95% CI)	47.9 (41.8, 54.0)	45.9 (39.8, 52.0)	51.8 (45.6, 57.9)	52.5 (46.4, 58.6)	51.8 (45.6, 57.9)
**Positive likelihood ratio (PLR),** (95% CI)	1.06 (0.70, 1.42)	0.85 (0.62, 1.09)	1.00 (0.71, 1.29)	0.96 (0.63, 1.29)	0.82 (0.46, 1.19)
**Negative likelihood ratio (NLR),** (95% CI)	0.96 (0.71, 1.21)	1.14 (0.88, 1.39)	1.00 (0.77, 1.24)	1.03 (0.76, 1.31)	1.15 (0.83, 1.47)
**C statistic,** (95% CI)	0.51 (0.44, 0.59)	0.54 (0.47, 0.60)	0.50 (0.44, 0.57)	0.51 (0.43, 0.58)	0.540 (0.46, 0.63)

[1] See eTable 2 for details about the calculation of performance characteristic values from a 2 x 2 table. Note that NEST ≥30 represents the base case reported in the manuscript and shown in Figure 1.

CI: confidence interval, NEST: Needs at the End-of-Life Screening Tool

© 2022 Cox CE et al. *JAMA Network Open*.

eTable 4. Comparison of family member survey responses to NEST scores

Outcome measure	**Value** n=257 (100%)	***p***
**Goal-concordant care reported**		0.365[1]
No	25.0 (13.0, 39.0)	
Yes	22.0 (11.0, 37.0)	
**Quality of communication**	-0.66	<0.001[2]
**How would you describe your relationship with the ICU doctors?**		<0.001[3]
Excellent	15.0 (8.0, 24.0)	
Good	28.0 (19.0, 42.5)	
Acceptable	45.0 (30.0, 55.0)	
Poor	79.0 (61.0, 95.0)	
**Do you expect your loved one to survive the hospitalization?**		0.266[3]
Almost certainly	21.5 (11.0, 31.5)	
Most likely	24.0 (12.0, 40.0)	
Probably not	17.0 (11.0, 42.0)	
Almost definitely not	32.0 (17.0, 37.0)	
**PHQ-9**	0.19	0.002[2]
**GAD-7**	0.19	0.003[2]
**PTSS**	0.19	0.002[2]
**IPC**		
Concern domain	-0.45	<0.001[2]
Decision making domain	-0.59	<0.001[2]
Discrimination domain	-0.13	0.044[2]

Values displayed as median NEST total score (interquartile range) or correlation of outcome with the NEST total score.
GAD-7: Generalized anxiety disorder 7-item scale, IPC: Interpersonal processes of care scale, NEST: Needs at the End-of-Life Screening Tool, PHQ-9: Patient health questionnaire 9-item depression scale, PTSS: Post-traumatic stress scale.
[1]Wilcoxon rank sum test, [2] Spearman correlation, [3] Kruskal-Wallis test

© 2022 Cox CE et al. *JAMA Network Open*.

eFigure. Percentage of participants reporting any level of individual need by trigger status

All $p>0.05$ for comparisons of individual items by trigger status by chi square tests.

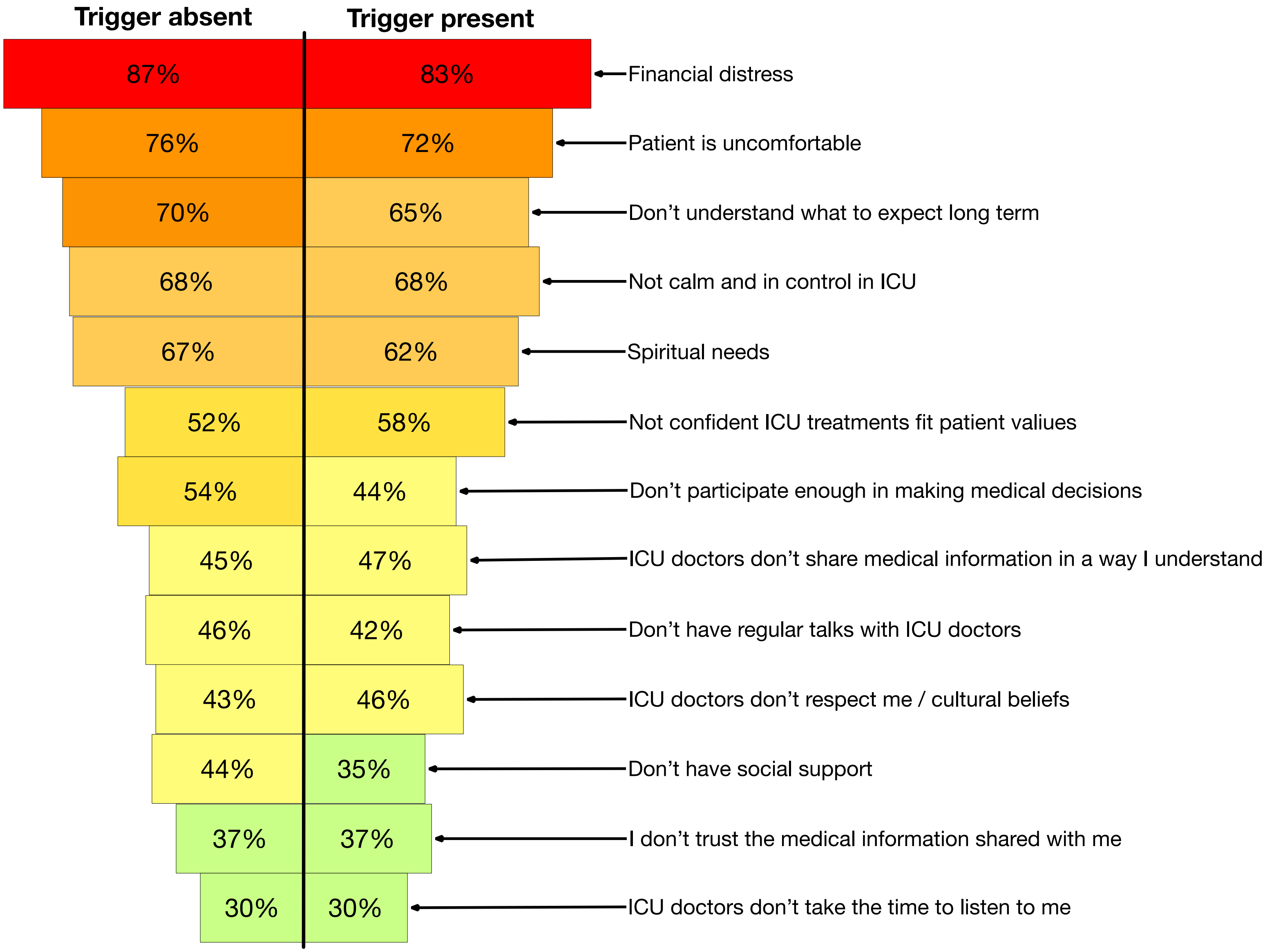

© 2022 Cox CE et al. *JAMA Network Open*.
